# Molecular markers for resistance against infectious diseases of economic importance

**DOI:** 10.14202/vetworld.2017.112-120

**Published:** 2017-01-25

**Authors:** B. M. Prajapati, J. P. Gupta, D. P. Pandey, G. A. Parmar, J. D. Chaudhari

**Affiliations:** Department of Animal Genetics and Breeding, College of Veterinary Science and Animal Husbandry, Sardarkrushinagar Dantiwada Agricultural University, Sardarkrushinagar - 385 506, Gujarat, India

**Keywords:** biomarkers, foot and mouth disease, genetic resistance, Johne’s disease, mastitis, tick and tick-borne diseases, tuberculosis

## Abstract

Huge livestock population of India is under threat by a large number of endemic infectious (bacterial, viral, and parasitic) diseases. These diseases are associated with high rates of morbidity and mortality, particularly in exotic and crossbred cattle. Beside morbidity and mortality, economic losses by these diseases occur through reduced fertility, production losses, etc. Some of the major infectious diseases which have great economic impact on Indian dairy industries are tuberculosis (TB), Johne’s disease (JD), mastitis, tick and tick-borne diseases (TTBDs), foot and mouth disease, etc. The development of effective strategies for the assessment and control of infectious diseases requires a better understanding of pathogen biology, host immune response, and diseases pathogenesis as well as the identification of the associated biomarkers. Indigenous cattle (*Bos indicus*) are reported to be comparatively less affected than exotic and crossbred cattle. However, genetic basis of resistance in indigenous cattle is not well documented. The association studies of few of the genes associated with various diseases, namely, solute carrier family 11 member 1, Toll-like receptors 1, with TB; Caspase associated recruitment domain 15, SP110 with JD; CACNA2D1, CD14 with mastitis and interferon gamma, BoLA­-DRB3.2 alleles with TTBDs, etc., are presented. Breeding for genetic resistance is one of the promising ways to control the infectious diseases. High host resistance is the most important method for controlling such diseases, but till today no breed is total immune. Therefore, work may be undertaken under the hypothesis that the different susceptibility to these diseases are exhibited by indigenous and crossbred cattle is due to breed-specific differences in the dealing of infected cells with other immune cells, which ultimately influence the immune response responded against infections. Achieving maximum resistance to these diseases is the ultimate goal, is technically possible to achieve, and is permanent. Progress could be enhanced through introgression of resistance genes to breeds with low resistance. The quest for knowledge of the genetic basis for infectious diseases in indigenous livestock is strongly warranted.

## Introduction

The livestock sector plays an important role in supporting the livelihood of livestock keepers, consumers, traders, and laborers worldwide. The livestock is an important segment of expanding and diverse agricultural sector of Indian financial system. Approximately, 70% people of this country are either directly or indirectly involved in the occupation related to agriculture and livestock rearing [1]. India’s huge livestock population is besieged by a large number of endemic infectious (bacterial, viral, and protozoan) and parasitic diseases. Infectious diseases in India cause an annual loss of INR 132 billion [2], mainly through morbidity, mortality, reduced fertility, production losses, and inefficient feed utilization. Some of the major infectious diseases having great economic impact on Indian dairy industries are mastitis, tick and tick-borne diseases (TTBDs), Johne’s disease (JD), tuberculosis (TB), foot and mouth disease (FMD), etc. Livestock owners and other stakeholders involved in promoting animal health can draw on a number of approaches to reduce negative effects of these diseases. The application of established methods for disease control, such as the use of antibiotics, chemotherapeutic agents, and the implementation of vaccinations protocols, most commonly had negative consequences by prompting variability among microorganisms and the appearance of drug-resistant strains, further leading to serious animal health-care problems [3-5].

Susceptibility to the mentioned infectious diseases suggested to have a genetic component. These ailments are good candidates for genetic selection as effective vaccine is either not available or if available, either costly or currently are at trial stage. Further, these diseases are difficult to cure and cause significant economic losses. Selective breeding, to increase resistance against infectious disease, will prove to be a low cost and sustainable practice. In livestock, a number of candidate genes have been studied and selected on the basis of their association to resistance or susceptibility in certain other diseases and their known role in disease pathogenesis. These genes include solute carrier family 11 member 1 (SLC11A1), interferon gamma (IFN-γ), peptidoglycan recognition protein 1 (PGLYRP-1), Toll-like receptors (TLRs), Caspase associated recruitment domain 15 (CARD15), mannose binding lectin-1 (MBL-1), nitric oxide synthase (NOS_2_), so on and so forth. Genome-wide association studies have attempted to confirm associations found and identify new genes involved in pathogenesis and susceptibility.

### Economic Losses

Among the major infectious diseases posing serious threat to Indian animal production systems, mastitis alone has remarkably rising impact where overall losses are estimated to be INR 71655.1 million [6]. Similarly, TB caused by *Mycobacterium bovis* has a wide host range and causes significant economic hardship for livestock farmers, with estimates of more than 50 million cattle infected worldwide [7]. JD is another dreaded disease and is responsible for economic losses of approximately US$ 250 million to the US dairy industry [8]. Although, economic loss due to JD have not been estimated in India but are supposed to have huge economic impact on livestock economy. Annual total economic loss due to FMD in India ranges from INR 12,0000 million to INR 14,0000 million [9]. On the other hand, ticks are able to transmit various pathogens and cause great damage to humans and animals worldwide [10]. In their rough estimation, Minjauw and McLeod [11] reported approximately 20000 million rupees per annum losses due to TTBDs in India. Further, some of these diseases are zoonotic and have a significant impact on public health especially among personnel who traditionally work with animals.

## Immuno-pathogenesis

### TB

Bovine TB (BTB) is a chronic infectious disease caused by *M. bovis*, characterized by the formation of granulomas in tissues and organs, more significantly in the lungs, lymph nodes, intestine and kidney including others. The entry of mycobacteria through mucous membranes leads to the recognition of bacterial cell wall components and the activation of inflammatory signaling pathways in phagocytes. The mycobacteria are then phagocytized by macrophages, and neutrophils are attracted to and accumulate at the site of initial infection. These cells interact with other cells involved in the innate and acquired immunological responses [12]. The host defense response to pathogens depends on the immune system.

Ingestion of the bacillus is followed by fusion of lysosomes with the phagosome to form phagolysosomes and it is there that the phagocytes attempt to destroy the bacillus [13]. However, virulent mycobacteria survive inside a mononuclear phagocyte by inhibiting phagosome fusion with pre-formed lysosomes, thereby limiting acidification. Protective immunity against mycobacterial infection is dependent on the activation of a cell-mediated immune (CMI) response. Inflammatory cytokines, i.e., interleukin 1 (IL-1), IL-2 and tumor necrosis factor alpha (TNF-α), produced by mononuclear cells sensitized by mycobacterial antigens recruit natural killer (NK) T-cells, CD4 T-cells, CD8 T-cells, and gamma delta T-cells [14]. Each of these cells produces cytokines that recruit additional cells to the site of infection resulting in the formation of granulomas and containment of infection [15]. Granuloma formation is an attempt of the host to localize the disease process. However, in the cases in which the host response in unable to destroy the bacillus due to condition that compromise immune function resulting in low CD4+ T-cell counts, such as old age, stress or HIV reactivation may occur, resulting in the release of bacilli and transmission of infection.

### JD

*Mycobacterium avium subspecies paratuberculosis* (MAP) is highly pathogenic mycobacteria affecting dairy cattle and other domestic ruminants globally [16]. The infection is usually acquired through the fecal-oral route, acquired early in life, often soon after birth, but clinical signs rarely develop in cattle below 2 years of age, because progression to clinical disease occurs slowly. After ingestion and uptake in the Peyer’s patches of the lower small intestine, this intracellular pathogen infects macrophages in the gastrointestinal tract and associated lymph nodes. It is possible that some animals may eliminate infection through a CMI response that encourages microbiocidal activity in macrophages, but the frequency with which this occurs is unknown.

The bacterium after crossing lumen of intestine is taken up by epithelioid macrophages which, once activated, elicit T-cell activation and clonal expansion [17]. Two T-helper cell subpopulations (TH_1_ and TH_2_) activate different host immune responses. *Mycobacterium paratuberculosis* infection appears to follow patterns similar to that of *M. tuberculosis*. These patterns entail an initial TH_1_ response (referred to as “tuberculoid”) that is characterized by a tissue infiltrate distinguished primarily by lymphocytes with few if any detectable organisms [18]. The TH_1_ response is also characterized by the production of the cytokine IFN-γ, one of the earliest detectable reactions to *M. paratuberculosis* infection, in addition to IL-2 and TNF-α. These cytokines are assumed to have a significant role in the CMI functions necessary to contain such an intracellular infection. During the early, subclinical stage of *M. paratuberculosis* infection, the TH_1_ T-cell activity appears to predominate. This subclinical phase of infection can last for months to years, as the bacilli are contained within macrophages and microscopic granulomas.

### FMD

The disease caused by FMD virus (FMDV), a member of the family Picornaviridae, is characterized by fever, profuse salivation, vesicles in the mouth and on the feet, and a drastic reduction in milk production. Sudden death in young stock may occur [19]. In cattle, primary replication site is nasopharynx and larynx epithelial tissues infected during the pre-viremic phase of the disease [20]. The mechanism, by which viral replication occurs without causing cell lysis, is not very much clear till today. In infected animals, high concentrations of virus can be found in lymph nodes, myocardium, lungs, and skin even in the absence of obvious lesions [21]. IL-10 is widely known for its anti-inflammatory response and to the inhibition of cellular responses *via* a variety of mechanisms [22]. Evidence also suggests that NK cells may be functionally defective during infection [23].

### TTBDs

Ticks are among the most competent and versatile vectors of pathogens and are only second to mosquitoes as vectors of a number of human pathogens, such as viruses, bacteria, rickettsia, and spirochetes, and the most important vector of pathogens affecting cattle worldwide [24]. The two genera of ticks - namely, *Rhipicephalus* and *Hyalomma -* are most widely distributed in India [25]. In general, the ticks become infected with the causative organism of diseases while feeding on infected animals. The infectious organism may be transmitted from stage to stage in the ticks or from the female ticks through the eggs to the larvae (transovarian transmission), resulting an increase of several thousand time in vector potential. When the next stage or generation subsequently feeds on another animal, the infectious organism is transmitted to that animal if it is susceptible to the disease.

Evidence suggests the role of both innate and adaptive immune mechanisms against TTBD. While major histocompatibility complex (MHC-I) restricted cytotoxic T-lymphocyte (CTL) responses have been observed in *Theileria parva* and *Theileria annulata* infected cattle, direct evidence for the existence of CTL activity against other tick-borne pathogens is still missing. Recently, it is observed by many that CD4+ T cells play a key role in the development of protective immunity against the TTBD. IFN-c produced by CD4+ T cells and NK cells activates macrophages for an enhanced phagocytosis and cytokine and nitric oxide production. These molecules either kill or inhibit the growth of the parasites. It is also advocated that memory T-helper cells provide help for the synthesis of opsonizing anti-parasite immunoglobulin (Ig) G_2_ antibodies. On the other hand, the overproduction of cytokines, particularly tumor necrosis factor alpha, leads to an exaggeration of the S52 (types of IgE) clinical symptoms and pathological reactions associated with TTBD [26].

In India, tropical theileriosis and babesiosis are the major tick-borne infections. In babesioses, sporozoites are injected into the host and directly infect erythrocytes, where they develop into piroplasms resulting in two or sometimes four daughter cells that leave the host cell to infect other erythrocytes [27] that lead to hemoglobinemia, hemoglobinuria and fever. This may be so acute as to cause death within a few days, during which the packed cell volume falls below 20% leading to anemia [28]. *Theilerioses* caused by *Theileria* species, where sporozoites injected by the ticks, which then enter lymphocytes and develop into schizonts in the lymph node draining the area of attachment of the tick, usually the parotid node. Infected lymphocytes are transformed to lymphoblasts which continue to divide synchronously with the schizonts so that each daughter cell is also infected [29]. Eventually, some schizonts differentiated into merozoites are released from the lymphoblasts and invade erythrocytes which lead to development of anemia. The dominating pathological lesion is generalized lymphoid proliferation resulting from uncontrolled proliferation of T-lymphocytes containing schizonts. The severe lymphocytolysis often leads to immunosuppression.

### Mastitis

In dairy industry, intramammary infections are among the most important diseases of cows that cause great economic losses [30]. Mastitis is a response of udder to different internal and external factors [31], and substantially, affects the milk quality and production of dairy cows. Bacterial pathogens are major threat to mammary gland, which causes irritation and pathological changes in mammary tissue. The degree of changes depends on the pathogenicity of bacteria and the inflammatory response [32]. Subclinical mastitis is 15-40 times more common than the clinical form [33]. The important major mastitis pathogens are *Escherichia coli*, *Staphylococcus aureus*, and *Streptococcus agalactiae* [34].

Following entry of bacteria into the mammary gland, large numbers of neutrophils migrate from the blood into the milk to control infection. This relies on the appropriate expression of genes, such as receptors present on cell surface as CD14 and TLRs, triggering a diverse array of responses including the early release of immune effector molecules and chemokines, facilitating the appropriate influx of cells following infection [35].

The genes involved (cytokines, receptors, etc.) in the pathogenesis of these diseases may be exposed for possible variation which may further be associated with disease susceptibly or resistance and may be explored for variations, which can be exploited for selection for resistance/susceptibility. Since the unraveling of the genetic material and the rapid development of molecular genetics tools, the last 50 years has witnessed a tremendous growth in the knowledge base and utilization of genetic information in tackling human and animal diseases. The genetic material in livestock animal species harbors a huge collection of genetic variations, these variations are usually in the form of deletions/insertions of nucleotides, single nucleotide polymorphisms (SNPs), gene duplications, copy number polymorphisms, e.g., variable number of tandem repeats (VNTRs) and microsatellites [36].

### Marker: A Potential Selection Tool

A genetic marker is a gene or DNA sequence with a known location on a chromosome and associated with a particular gene or trait. It is a variation, which may arise due to mutation or alteration in the genomic loci that can be observed [37]. Markers may be morphological, which uses phenotype of an animal for a specific trait as a tool for selection [38]. It may be chromosomal which utilizes karyotypes, bandings, repeats, translocations, deletions, and inversions to investigate the genetic resources of animals [39]. It may also be biochemical, such as the blood type and isozymes which represent biochemical traits that could be analyzed by protein electrophoresis. The most advanced of these markers are molecular markers which are based on the nucleotide sequence mutations within the individual’s genome; they are the most reliable markers available [39].

The first approach in application of molecular markers has been the use of candidate genes. It is assumed that a gene involved in a certain trait could show a mutation causing variation in that trait, and any variations in the DNA sequences, that are found, are tested for association with variation in the phenotypic trait [40]. Till now, many types of molecular markers have been utilized to detect the variation among individual and population. These markers can be classified into various types.

### Restriction fragment length polymorphism (RFLP)

The RFLP is defined by the existence of alternative alleles associated with restriction fragments that differ in size from each other. RFLP is the nucleotide base substitutions, insertions, deletions, duplications, and inversions within the whole genome, can remove or create new restriction sites [39]. RFLP analysis is useful to find out where a specific gene for a disease lies on a chromosome [41].

### Single-strand conformation polymorphism (SSCP)

The SSCP technique, discovered by Orita *et al*. [42], has been found to be very useful for quick, sensitive and relatively inexpensive detection of differences in the nucleotide sequences of closely related genomes.

### Simple sequence repeats (SSR)/microsatellites

SSR loci, referred as VNTRs and simple sequence length polymorphisms, are found throughout the nuclear genomes of most eukaryotes and to a lesser extent in prokaryotes [37]. Microsatellites range from one to six nucleotides in length and are classified as mono-, di-, tri-, tetra-, penta-, and hexanucleotide repeats. They are tandemly repeated (usually 5-20 times) in the genome with a minimum repeat length of 12 base-pairs. The number of repeats is variable in populations of DNA and within the alleles of an individual [43].

### SNP

In 1996, Lander proposed a new molecular marker technology named SNP [44]. When a single nucleotide in the genome sequence is altered, this will represent the SNP. In other words, it refers to a sequence polymorphism caused by a single nucleotide mutation at a specific locus in the DNA sequence [37].

### Association of Markers with Disease Resistance or Susceptibility

Advances in molecular genetics are taking place leap and bound, and with the advent of new technology, detection of variation in markers is becoming easier with high precision. Among the more recent technology of detection of polymorphism is the real-time polymerase chain reaction (RT-PCR) and its use in establishing association with comparison of m-RNA expression in different pathological conditions.

### Markers associated with mastitis

Deb *et al*. [45] from their study of SNP detection by RT-PCR in bovine CACNA2D1 gene (chromosome 4) at G519663A locus, demonstrated that GG genotype is associated with the lowest somatic cell score (SCS), and the transcript abundance of CACNA2D1 mRNA and was favorable for the mastitis resistance. Similar finding with the help of PCR-RFLP was observed by Yuan *et al*. [46] where he analyzed the SNPs of CACNA2D1 gene and associated it significantly with milk SCS. Yuan *et al*. [47] with the help of PCR-RFLP reported the combined genotype analysis of three SNPs (G22231T, T25025A and C28300A in bovine BRCA1 gene) and showed association of BBDDFF genotype with the highest SCS that indicated mastitis susceptibility. However, AACCEE genotype associated with the lowest SCS was favorable for the mastitis resistance. Asaf *et al*. [48] reported polymorphism of the BRCA1 SNPs, i.e., G22231T but reported lack of association of allelic variants with mastitis susceptibility in Vrindavani cattle by PCR-RFLP. Wang *et al*. [49] with PCR-SSCP identified A-G SNP at nucleotide 4525 in intron 1 of TLR4 gene and concluded that this variation might play an important role in bovine mastitis resistance and with the help of PCR-RFLP Deb *et al*. [50] observed the SNP (g9788 C>T) where CC genotype was more prevalent than TT among Frieswal crossbreed cattle, and C allele was observed to be favorable for mastitis resistance and may be used as one of the molecular marker. RT-PCR analysis of C4A gene (chromosome 8) could reveal the SNP rs132741478: g.2994A>G in the coding sequence of the ß-chain and is found to be related with mastitis resistance [51].

Liu *et al*. [52] using PCR-SSCP reported the association of the ATP1A1 (chromosome 3) polymorphism with the mastitis trait in Holstein cows, indicating that genotype CC of ATP1A1 was related to mastitis resistance, whereas genotype CA was related to mastitis susceptibility. PCR-RFLP study on SNPs of bovine PGLYRP-1 (chromosome 18) identified a total of ten SNP loci. The association study showed that T-35A, T-12G and G-102C were significantly associated (p<0.05) with SCS [53]. PCR-RFLP with *Hinf*I enzyme, of CD14 gene (Chromosome 7) by Selvan *et al*. [54] in Karan Fries cows revealed three genotypes CC, CD and DD, that differed significantly regarding mastitis incidence. CC genotype was found to be more resistant when compared with CD and DD. On the same line with the help of PCR-SSCP, Gupta *et al*. [55] reported a non-synonymous SNP in exon two of CD14 gene and out of three genotype observed BB genotype was found to be more susceptible to mastitis.

Asaf *et al*. [56] in their work on MBL-1 (Chromosome 28) with PCR-RFLP claimed no significant association between SNP “rs109231409” with mastitis tolerance or susceptibility in Vrindavani crossbred cattle. Further, Zhao *et al*. [57] with correlation analysis showed that the g.1197 C>A marker was significantly correlated to SCS, and the g.1164 G>A locus had significant effects on SCS, suggesting possible roles of these SNPs in the host response against mastitis in Chinese Holsteins.

In microsatellite study, Guo *et al*. [58] analyzed four microsatellite DNA loci BM1818, BM1258, BM1443, and BM1905 associated with the somatic cell counts (SCC) in cow milk for genetic variation in 240 Beijing Holstein cows and found that BM1818, BM1258, BM1443, and BM1905 were having respectively 4, 5, 8 and 6 alleles. The allele size ranges for BM1818, BM1258, BM1443 and BM1905 were 274-286, 92-106, 154-170 and 187-201 bp, respectively. Chu *et al*. [59] analyzed genetic variation of seven microsatellite loci BM1818, BM1258, BM1443, BM1905, BM302, BM4505 and CYP21 which were closely linked to SCS in 240 Beijing Holstein cows and found a significant association with the SCS. Based on association with SCC, Ranjan [60] detected significant marker allele affecting the incidence of mastitis for markers DIK20, BM302, BM4505, CYP21 and BMS2684 in crossbred cattle. Gupta [61] based on association with SCS detected significant microsatellite marker allele effect on the incidence of mastitis for marker BM1818 and BM1443 in Vrindavani crossbred cattle maintained at Indian Veterinary Research Institute, Bareilly, Uttar Pradesh.

### Markers associated with TB

In bovine SLC11A1 gene (chromosome 2), Cheng *et al*. [62] with the help of direct sequencing of PCR products reported a polymorphism in intron 4 at g.107117369C>T and in exon 4 at g.107117166A>G which results in change of an amino acid from alanine to threonine and concluded that these two polymorphisms may contribute to SLC11A1-mediated BTB susceptibility. Further, Baqir, *et al*. [63] detected a polymorphism in SNP rs109915208 in the same gene, in Indian cattle population, revealed three genotypes CC, CT and TT, of which animals having “CT” genotype and “T” allele were resistant to TB. Bovine CARD15/NOD_2_ (chromosome 18) in cattle plays an important role in TB resistance. Wang *et al*. [64] with the help of PCR-RFLP products reported a polymorphism the TGGACA and CAGACA that were significantly different between cases and controls (p<0.05). Cattle with these two haplotypes have greater possibility to be infected and attacked by *M. bovis*. Further, Qin *et al*. [65] investigated the polymorphism of *Sty*I enzyme cut site in *CARD*15 gene and obtained SNP was found to be significantly associated with the susceptibility to TB of Yunnan plateau dairy cows. Cheng *et al*. [66] observed NOS_2_ gene (chromosome 19), g.19958101T>G polymorphism by PCR-RFLP and concluded that this gene may contribute to the susceptibility of TB in cattle.

Sun *et al*. [67] performed PCR-SSCP and PCR–RFLP, detected G1596A polymorphism in the TLR1 gene and found it to be associated with BTB infection status in Chinese Holstein cattle. Alfano *et al*. [68] detected the SNP (1650 G>A) located in the TLR2 gene with the help of PCR-direct sequencing, which is associated with disease resistance in water buffalo. The CC genotype at the TLR4 672 A>C locus polymorphism had significant association with resistance to TB in water buffalo and the TT genotype at TLR9 2340 C>T locus had significant association with susceptibility to TB in water buffalo [68].

Among the microsatellite Markers, as per the observation of Zanotti *et al*. [69] the genotype 152/154 of BM644 microsatellite was significantly associated with susceptibility to TB, while 267/270 of AR028 microsatellite with resistance. Kadarmideen *et al*. [70] from their work on African zebu cattle reported that alleles of 211 and 215 bp were found to be associated with TB resistance. Ali *et al*. [71] reported that the effect of alleles in ILSTS006 and BM2113 markers on BTB phenotypes were significantly associated with decreasing BTB disease incidence in cattle.

### Markers associated with JD

Ruiz *et al*. [72] performed SNPlex genotyping and found that the GTCC haplotype in CARD15 gene (chromosome 18) carries the C allele for the SNP c.1908C>T and it was significantly associated with susceptibility to MAP infection in Spanish Holstein-Friesian and Dutch Holstein-Friesian. Further, Pinedo *et al*. [73] in their study concluded that amino acid substitution in C733R (2197T>C) appears to be associated with susceptibility to paratuberculosis infection in the Brahman-Angus breed of cattle by RT-PCR method. Ruiz *et al*. [74] again using Taqman RT-PCR assay found that the SNP c.587A>G (N196S) in exon 5 of the bovine SP110 gene (chromosome 18) is associated with the susceptibility to MAP infection in dairy cattle. Similarly, with the help of RT-PCR, Koets *et al*. [75] reported that the TLR2-1903 T/C SNP was significantly associated with resistance to MAP in Holstein-Friesian cows. They further reported that SNP c.1707T>C in a Dutch Holstein-Friesian population was highly suggestive of susceptibility to MAP infection in cattle. Ruiz *et al*. [74] found that the TLR4 226G>C SNP has direct implication of causal variant in susceptibility to paratuberculosis in HF cattle. Kumar [76] reported that A>G and C>T in TLR-4 gene are associated with the paratuberculosis in cattle. Pinedo *et al*. [73], with the help of direct sequencing, revealed significant association of MAP infection for SNP (2781G>T) *IFN-γ* gene in Holstein, Jersey and Brahman-Angus crosses.

The microsatellite analysis done by Pinedo *et al*. [77] revealed five different alleles (sizes 273, 275, 277, 279 and 281 bp). Further, it was observed that there was significant difference in allelic frequencies between cases and controls for SLC11A1 microsatellite (p=0.002) indicating an association between infection and SLC11A1 alleles.

### Markers associated with FMD

Singh *et al*. [78] in their study with ARMS-PCR method concluded that SNP G29A mutation in the 5 UTR of the ITGB6 gene (chromosome 2) associated with resistance to FMD infection in the zebu cattle. DNA sequence analysis revealed eleven different alleles in the Hariana cattle population and it was observed that DRB3 alleles *0201, *0801 and *1501 always ranked high for protective immune response whereas alleles *0701, *1103 and *1101 consistently ranked low for unprotected immune response for all the serotypes of FMDV [79]. Similarly, with the help of Hemi-nested PCR-RFLP, Lei *et al*. [80] reported 6 RFLP patterns of BoLA-DRB3 (167/65/48/4, 167/65/52, 219/65, 167/65/52/48/4, 219/65/48/4, 219/65/52 bp) and named AA, BB, CC, AB, AC and BC. CC and BC genotype were actually associated with resistance to FMD by contrast AA genotype was associated with susceptibility to FMD in Wanbei cattle. Chinsangaram *et al*. [81] with sequence analysis reported the IFN-α/β is an ideal candidate for rapid induction of FMDV resistance in animal.

### Markers associated with TTBDs

Recent years have witnessed an emergence of various dreaded TTBDs (CCHF, etc.) and lack of its effective control measures has compelled to look for certain markers to be included in selection programs for controlling the menace of TTBDs. IFN-γ is one such gene (chromosome 5) in which Maryam *et al*. [82] with sequence analysis reported the SNP (2051C>A) and found that it was associated with tick resistance in Sahiwal cattle. Similarly, Martinez *et al*. [83] suggested that BoLA-DRB3.2 (Chromosome 23) alleles association between BoLA marker-DRB 3.2allele, 18, 20, and 27 for lower tick numbers in a reference Holstein × GYR F2 population in Brazil can be used to help in the selection of animals resistant to tick infestation. Rodriguez *et al*. [84] reported that the allele 128bp of DRBP1 locus and allele 152 bp of BM1815 may be involved in susceptibility to tick infestation in Holstein × Zebu animals. Dewangan *et al*. [85] on the basis of comparative fold change analysis of m-RNA concluded that there was a significant up-regulation in SIRPA, PRNP, and MHC DQα genes and significant down-regulation in TLR10, cMAF and MAFB genes in *T. annulata* infection in crossbreds as compared to indigenous cattle. Recently in a study of global gene expression profile in PBMCs with the help of Microarray, NFKBID, BoLA-DQB, HOXA13, PAK1, TGFBR2, NFKBIA genes showed breed specific differences associated with *T. annulata* infection [86].

### Limitations and Future Scope

One of the major questions which trouble all of us is what to do with large number of sporadic SNPs studies with small number of samples? Are these studies baseless or they can still be made useful? Recently, Schaub *et al*. [87] using encode consortium, identified functional SNPs which may be associated with the disease phenotypes in 80% of the previously reported “possible associations.” Experimental statistician can stratify these studies accepting to various attributes and can join them in a meta-analysis format to make the studies more robust. Moreover, these small studies may pave the way for a bigger and more robust study. In that case, such small studies are like mini pilot studies spread in time and space. However, we also have to understand that no amount of statistical jugglery can hide the weakness from the basic design of the study. Hence, there is need to explore more and more SNPs in our population and establish its association with diseases but it is also expected that all SNP-based studies should be carefully crafted and well-designed from the beginning so that the utility of that particular study will exist at sent for some time.

## Conclusion

The evidence presented above indicates a detectable genomic basis of the various infectious diseases of economic importance, and there is a strong case for the inclusion of genetic elements within disease control strategies, particularly in the light of constraints to the sustainability of other classical methods. Disease resistance traits are among the most difficult to include in the classical “Biometrical” farm animal genetic programs. A further complication is that disease resistance is most often an “all or none” trait. Although there may well be quantitative polygenic variation in resistance status, the observations are often limited to “Sick or healthy.” This, too, reduces heritability compared with other polygenic traits, such as milk production. There are many documented instances of breed and individual differences in genetic disease resistance among farm animals. The current studies in cattle for selection against mastitis and JD in India have shown some encouraging results and have motivated the scientist and workers working in this area to look for more and more variations and associate these with disease resistance or susceptibility. Thus, selection for genetic disease resistance provides a potential avenue for improving the health status of farm animals, increasing productivity and reducing the need for pharmaceutical intervention, thereby reducing cost and delaying the appearance of resistant pathogens.

Breeding for genetic resistance is one of the promising ways to control the infectious diseases. High host resistance is the most important method for controlling such diseases, but no breed is totally resistant. Total resistance to these diseases is the ultimate goal, is technically possible to achieve, and is permanent. Progress could be enhanced through introgression of resistance genes to breeds with low resistance. The quest for knowledge of the genetic basis for infectious diseases in livestock is strongly warranted, given that 70 % of people are either directly or indirectly earning their livelihood in India by livestock based occupation.

## Authors’ Contributions

BMP prepared the initial version of the manuscript. GAP and JDC assisted in literature collection. JPG and DPP conceived the idea, revised the manuscript and made final critical scientific corrections. All authors read and approved the final manuscript.
